# Supplementary material for: Versatile bubble maneuvering on photopyroelectric slippery surfaces
Source: Nat Commun. 2023 Oct 3;14:6158. doi: 10.1038/s41467-023-41918-y (PMC10547833; doi:10.1038/s41467-023-41918-y)
Supplement: Supplementary file 1 — Supplementary Information [file 41467_2023_41918_MOESM1_ESM.pdf]

Supplementary Information for

Versatile bubble maneuvering on photopyroelectric slippery surfaces

Haiyang Zhan[1], Zichao Yuan[1], Yu Li[1], Liang Zhang[1], Hui Liang[2], Yuhui Zhao[2,3], Zhiguo Wang[2,3], Lei Zhao[1], Shile Feng[1], Yahua Liu[1]*

[1]State Key Laboratory of High-performance Precision Manufacturing, Dalian University of Technology, Dalian 116024, P. R. China

[2]Shenyang Institute of Automation, Chinese Academy of Sciences, Shenyang 110016, P. R. China

[3]Institutes for Robotics and Intelligent Manufacturing, Chinese Academy of Sciences, Shenyang 110016, P. R. China

*Corresponding author: yahualiu@dlut.edu.cn (Y.L.)

This PDF file includes:

Supplementary Figures 1 to 16

Supplementary Discussions 1 to 5

Supplementary References

Other Supplementary Materials for this manuscript include the following:

Supplementary Movies 1 to 9

Supplementary Figures

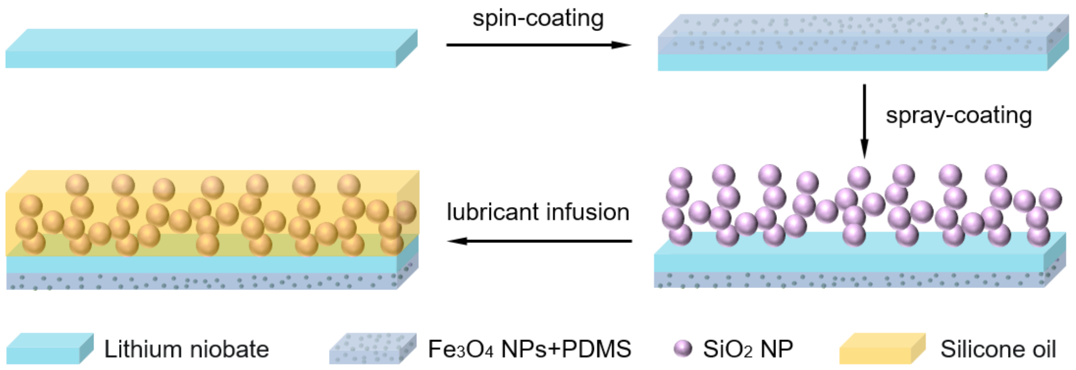

Supplementary Figure 1 | Schematics showing the preparation procedure of the PESS.

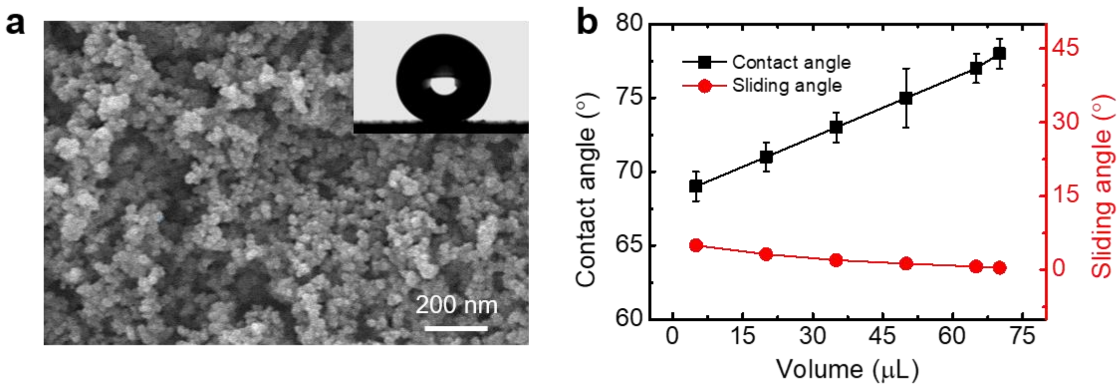

Supplementary Figure 2 | **Characterization of the PESS. a** SEM image of the PESS before being perfused with silicone oil. **b** The variation of bubble contact angle and sliding angle on the PESS with bubble volume. The error bars of the data denote the standard deviation of three measurements. Source data are provided as a Source Data file.

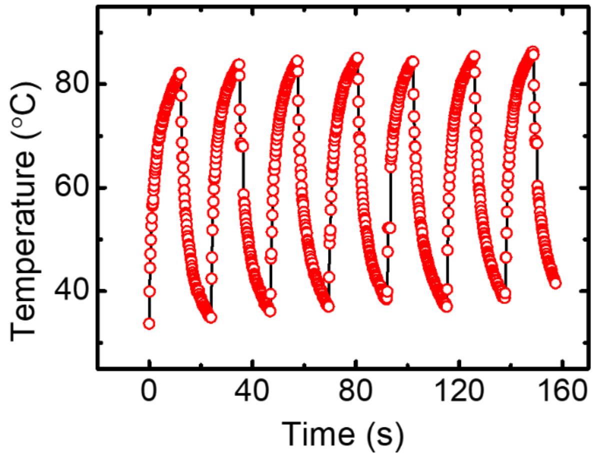

Supplementary Figure 3 | Cyclic stability of heating performance of the PESS. Source data are provided as a Source Data file.

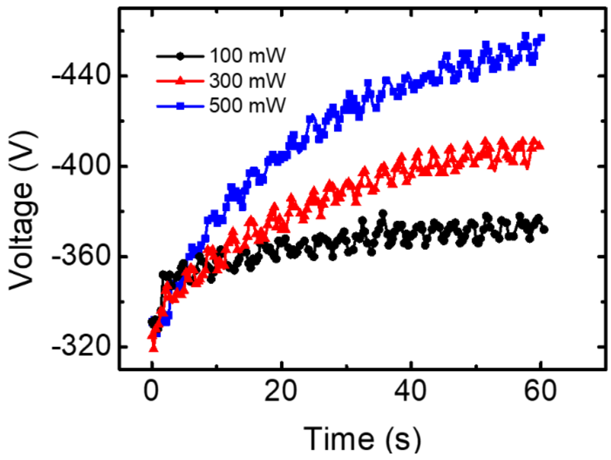

Supplementary Figure 4 | Surface voltage as a function of time. Here, the surface voltage was measured by an electrometer. Source data are provided as a Source Data file.

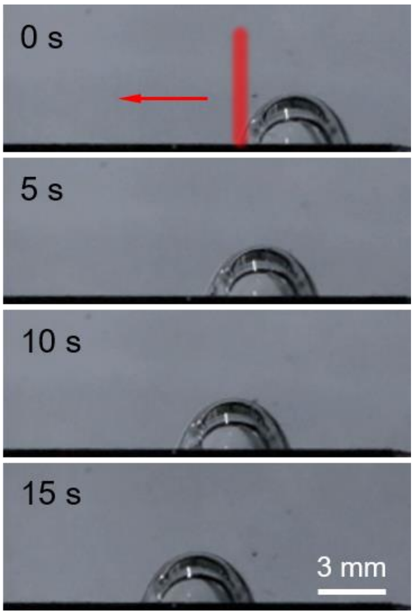

Supplementary Figure 5 | The transportation of a 35-μL bubble on the PSS under NIR irradiation. The bubble moves to the left.

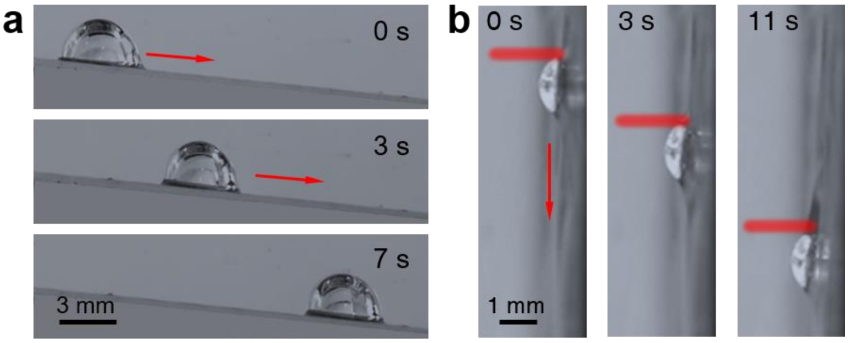

Supplementary Figure 6 | The anti-buoyancy movement of bubbles on the PESS. **a** Sequential images showing a 35-μL bubble transports against buoyancy at 2 mm s^{-1} on the PESS with an inclination of ~ 7°. **b** Sequential images showing the bubble vertical downward movement on the PESS with a lubrication layer viscosity of 100 mPa s under NIR irradiation.

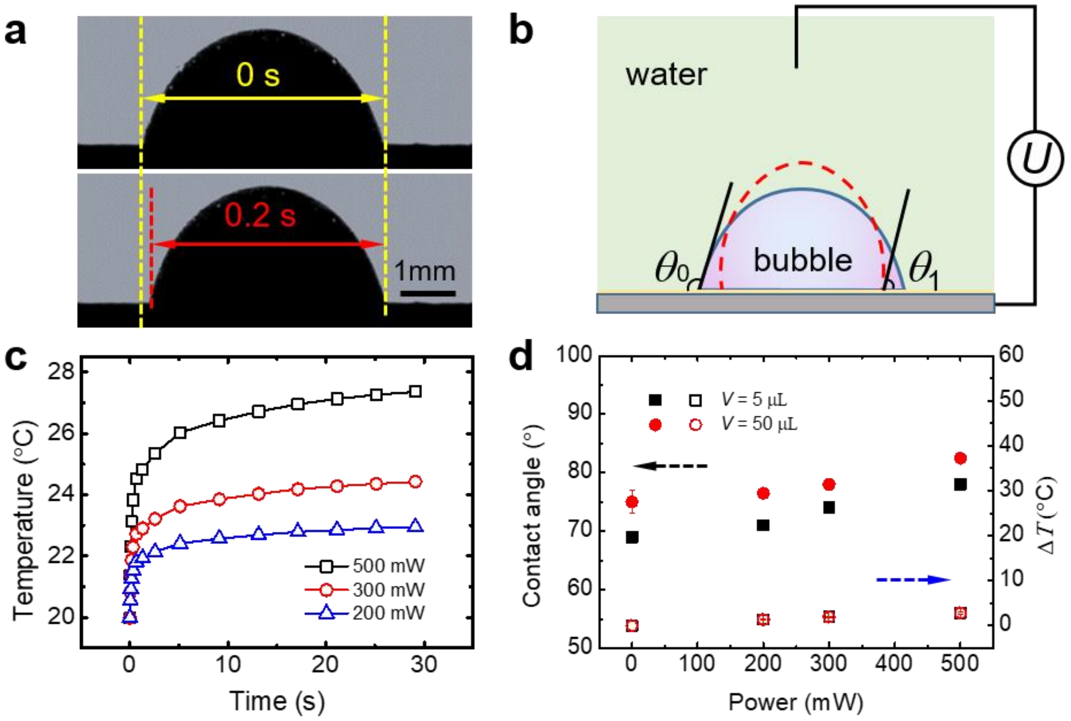

Supplementary Figure 7 | The mechanism of bubble manipulation on the PESS. a Light-induced bubble deformation on the PESS. **b** Schematic showing the variation of bubble contact angle under the electrowetting-on-dielectric effect. **c** The temperature variation as a function of irradiation time with varying laser power. **d** The variation of bubble contact angle and temperature difference at the three-phase contact line with the laser power. Source data are provided as a Source Data file.

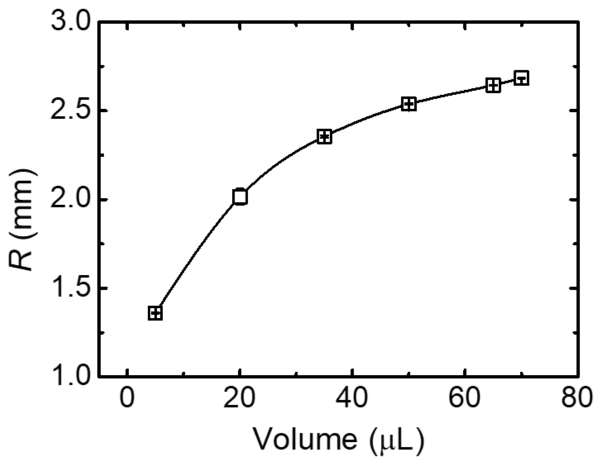

Supplementary Figure 8 | The variation of the base radius of the bubble with its volume on the PESS. The error bars of the data denote the standard deviation of two measurements. Source data are provided as a Source Data file.

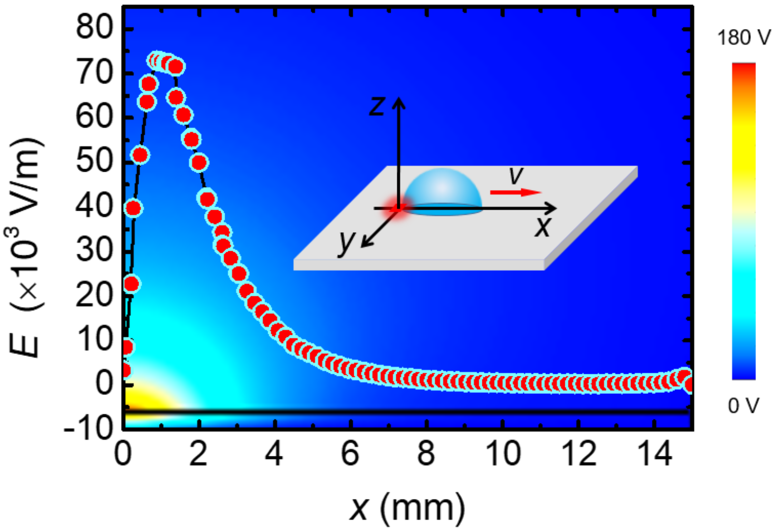

Supplementary Figure 9 | Variation of electric field intensity with distance. The insert shows the coordinate system established with the spot center as the origin. Source data are provided as a Source Data file.

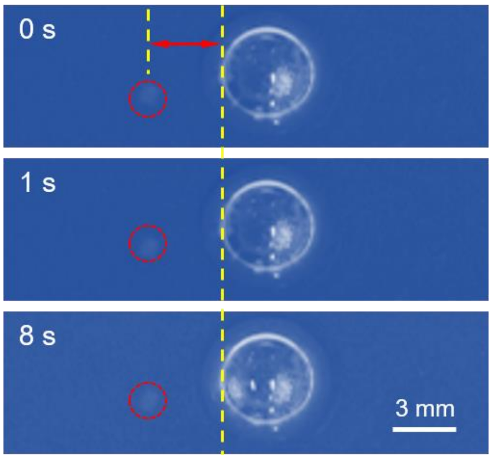

Supplementary Figure 10 | Sequential images showing the bubble movement under the irradiation point 3 mm away from the left end of the bubble on the PSS.

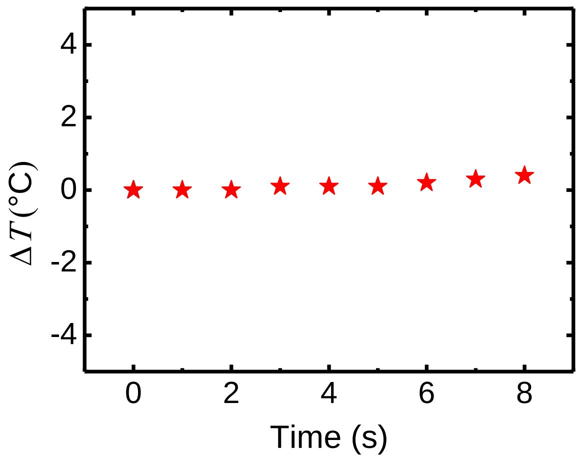

Supplementary Figure 11 | The variation of temperature difference with irradiation time under the irradiation point 3 mm away from the left end of the bubble on the PESS. Source data are provided as a Source Data file.

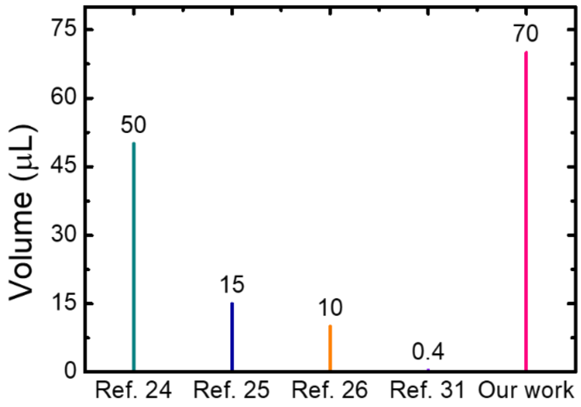

Supplementary Figure 12 | The bubble size that can be precisely manipulated on different surfaces. Source data are provided as a Source Data file.

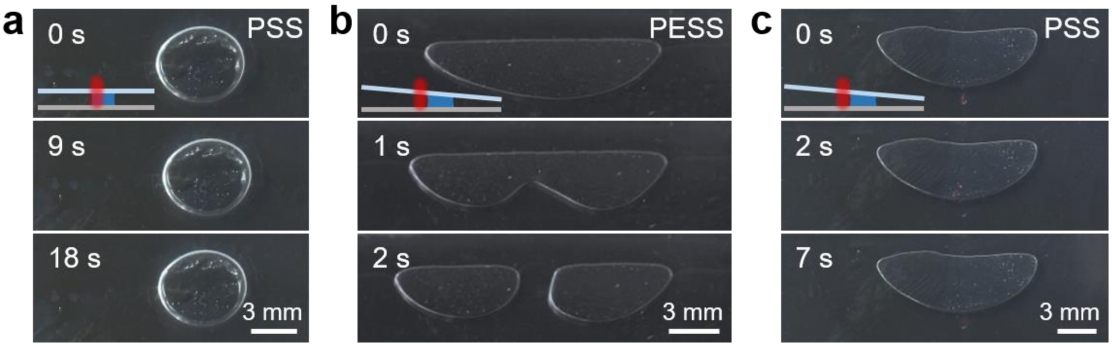

Supplementary Figure 13 | Bubbles manipulation on the PESS and PSS. a Sequential images showing the movement of compressed bubbles on the PSS under NIR irradiation. **b, c** Splitting of compressed bubbles by a slanted transparent slippery surface on the PESS (**b**) and PSS (**c**), respectively.

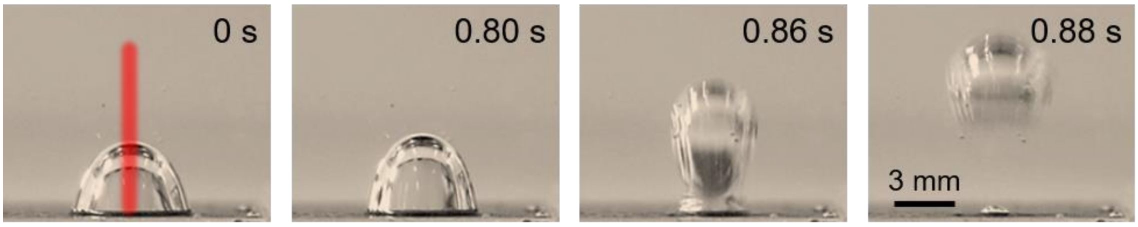

Supplementary Figure 14 | Sequential images showing the bubble detachment from the PESS under NIR irradiation.

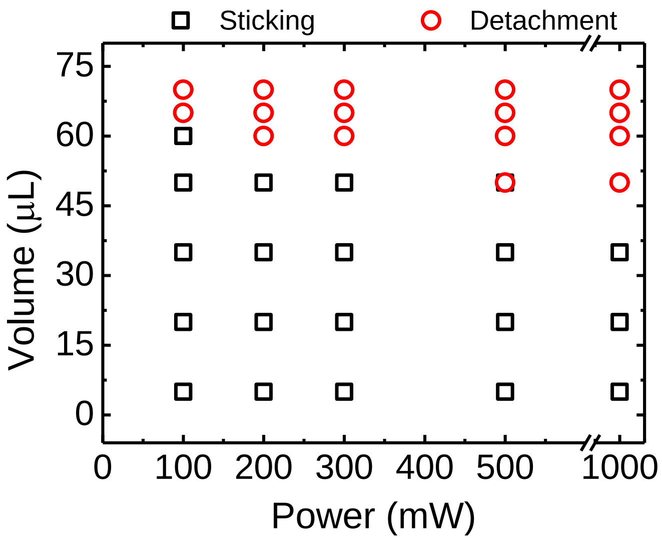

Supplementary Figure 15 | Phase diagram showing the diverse behaviors of bubbles with different sizes under various laser power. Source data are provided as a Source Data file.

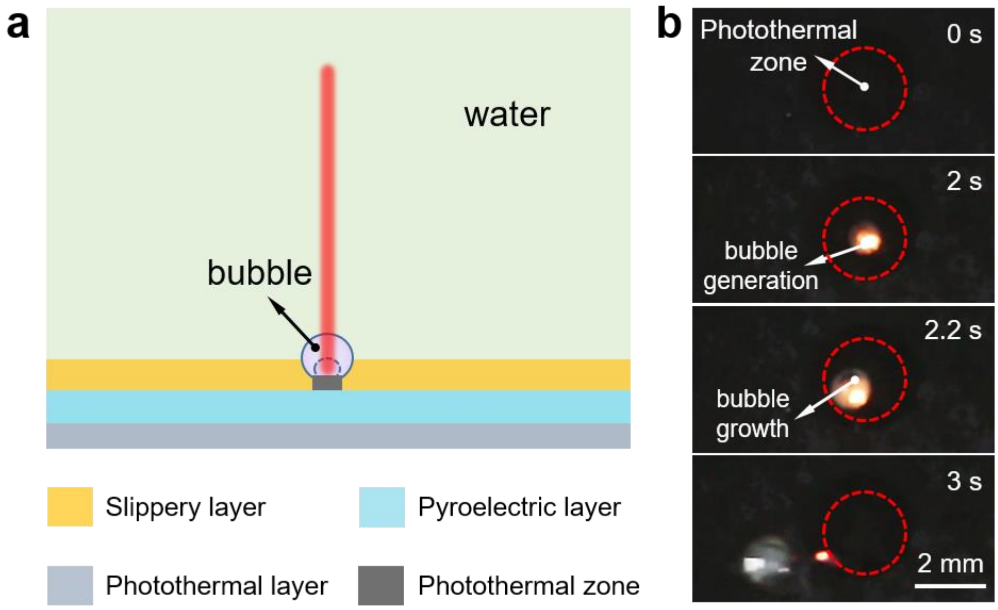

Supplementary Figure 16 | Surface bubble generation by leveraging the photothermal effect. a Schematics showing the mechanism for surface bubble generation. **b** Sequential images showing the generation of a surface bubble under NIR irradiation. Specifically, dispersed localized photothermal zones can be produced from the mixture of Fe_3O_4 nanoparticles and PDMS, which is sandwiched between the slippery layer and the pyroelectric layer during the preparation process of the PESS. When the photothermal zone is irradiated by the NIR light, part of the light energy is converted into heat energy and thus a bubble is generated due to the photothermal effect[1]. Note that, this method is time-consuming and difficult to accurately control the bubble size.

Supplementary Discussion 1. Bubble transport on the PSS

As reported previously[2], the bubble transportation on photothermal slippery surfaces under NIR irradiation is attributed to the Laplace force generated by the NIR-induced asymmetric deformation of bubbles. Initially, the left and right contact angles of the bubble on the PSS are equal to each other without irradiation as $\theta=\theta_{a0}=\theta_{b0}$ (Supplementary Fig. 17), which can be expressed by

$$\cos\theta = \frac{\gamma_{OL}-\gamma_{OG}}{\gamma_{LG}} \tag{S1}$$

where γ_{OG}, γ_{OL} and γ_{LG} are the tensions of the oil-gas, oil-liquid and liquid-gas interfaces, respectively. When the NIR light irradiating on the left side of the bubble, the temperature of the illuminated position rises quickly, which leads to a decreased γ_{OG}, corresponding to a decreased θ_{a0} while θ_{b0} remains relatively unchanged (Supplementary Fig. 17) and an asymmetric deformation of the bubble. This asymmetric deformation of the bubble generates a Laplace force $F_L=2R\gamma_{LG}(\cos\theta_{a1}-\cos\theta_{b0})$ that drives the bubble to chase the light source. By contrast, the surface tension change can be neglected on the PESS due to the low temperature rise.

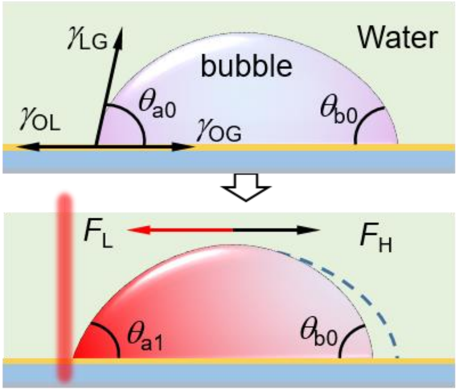

Supplementary Figure 17 | Schematics showing the force analysis in NIR-induced bubble transport on the PSS.

Supplementary Discussion 2. Numerical study of the electric field

In order to analyze the electric field distribution on the surface illuminated by the NIR light, we first performed a finite element analysis of the temperature variation. When the NIR light passes through the slippery layer and the thermoelectric layer, it reaches the photothermal layer. The photothermal effect of Fe_3O_4 nanoparticles will rapidly increase the temperature of the photothermal layer, and accordingly, the temperature of the pyroelectric crystal rises rapidly due to thermal conduction. The temperature distribution of the lithium niobate crystal can be studied by using COMSOL Multiphysics 5.6. The

physical field chosen for the finite element analysis is solid-fluid heat transfer, and the heat conduction equation of solid could be expressed as

$$\rho_s c_s \frac{\partial T}{\partial t} - \nabla \cdot k_s \nabla T = S(r,t) \quad \text{(S2)}$$

where ρ_s, c_s and k_s denote the mass density, specific heat, thermal conductivity of the solid, respectively, and S is heat source. And the heat conduction equation of fluid is

$$\rho_l c_l (\frac{\partial T}{\partial t} + u \cdot \nabla T) - \nabla \cdot k_l \nabla T = S(r,t) \quad \text{(S3)}$$

where ρ_l, c_l and k_l denote the mass density, specific heat, thermal conductivity of the liquid, respectively, u is the velocity of fluid. The initial condition is $t = 0$, and $T = 293.15$K.

The heat source term of lithium niobate crystal is

$$S(r,t) = \frac{P\alpha_1\omega_1\omega_2}{\pi R_0^2 d_l} e^{-\frac{2(x^2+y^2)}{R_0^2}} \quad \text{(S4)}$$

where R_0 is the width of laser spot, d_l denotes the thickness of lithium niobate crystal, ω_1 is the transmittance of superomniphobic surface, ω_2 is the transmittance of the pyroelectric crystal, α_1 is the photothermal conversion coefficient of the photothermal film, and P is the laser power. In our case, $\omega_1 = 0.68$, $\omega_2 = 0.72$, $\alpha_1 = 0.99$, $R_0 = 0.5$ mm and $d_l = 0.5$ mm.

The electric field distribution was simulated by using COMSOL Multiphysics 5.6 with the governing equations expressed as

$$\boldsymbol{E} = -\nabla U \quad \text{(S5)}$$

$$\nabla \cdot (\varepsilon_0 \varepsilon_r \boldsymbol{E}) = \rho_V \quad \text{(S6)}$$

where $\boldsymbol{E}$ is the electric field strength, U is the electric potential, and ρ_V is charge density. The boundary condition can be expressed by surface charge densities as

$$\mathbf{n} \cdot (\boldsymbol{D_1} - \boldsymbol{D_2}) = P_c \Delta T \quad \text{(S7)}$$

where $\boldsymbol{D_1}$ and $\boldsymbol{D_2}$ are the electric displacement of lithium niobate crystal and water at the interface, respectively, $\mathbf{n}$ is the inward normal to the boundary, and P_c is pyroelectric coefficient of the pyroelectric crystal. The initial condition of electric potential can be set as $U = 0$ because the temperature rise $\Delta T = 0$ at first[3-5].

Supplementary Discussion 3. Dielectrophoretic force and bubble steady moving velocity

The electric field intensity needs to be determined in order to calculate the dielectrophoretic force F_{DX} along the moving direction, i.e., x direction. Here, the surface charge at the light spot is treated as a

point-charge when the NIR light irradiated on the PESS, and the electric field intensity induced by the point charge can be expressed as

$$E = K_e \frac{Q}{r^2} \tag{S8}$$

where $K_e = 9 \times 10^9$ N m^2 C^{-2} is Coulomb constant, and Q is the amount of charge. Therefore, F_{DX} can be rewritten by

$$F_{DX} = \int \frac{1}{2}\varepsilon_0(\varepsilon_A - \varepsilon_W)\nabla E_X^2 d\Omega \;\; = -2\varepsilon_0(\varepsilon_A - \varepsilon_W)K_e^2Q^2 \int r^{-5}\, d\Omega$$

$$\approx -\varepsilon_0(\varepsilon_A - \varepsilon_W)K_e^2Q^2 \int_a^{2R} \pi f^2(x) x^{-5}\, dx \tag{S9}$$

where ε_0, ε_A and ε_W are the permittivity of vacuum, the relative dielectric constant of air, and the relative dielectric constant of water, respectively, a is the distance of the bubble edge from the spot center which is close to zero (Supplementary Fig.18), and R is the base radius of the bubble on the PESS.

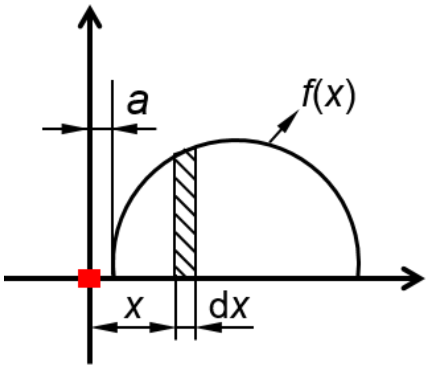

Supplementary Figure 18 | A reference system established with the spot center as the origin of coordinates.

Considering the sinusoidal shape of the bubble contour curve, i.e., $f(x) = R\sin(\pi x/2R)$, F_{DX} can be rewritten as $F_{DX} \approx -\pi\varepsilon_0(\varepsilon_A - \varepsilon_W)K_e^2Q^2 \int_a^{2R} x^{-5}R^2 \left(\sin\frac{\pi x}{2R}\right)^2 dx$, and an integration leads to

$$F_{DX} \approx D\Delta T^2 \left(\frac{3\pi^2}{48a^2} - \frac{4\pi^4+\pi^2}{192R^2}\right) \tag{S10}$$

where $D = -\pi\varepsilon_0(\varepsilon_A-\varepsilon_W)K_e^2P_c^2A^2$ is a constant and A is the area of the NIR spot. According to Equation (S10), F_{DX} increases with the temperature rise and bubble volume, which is consistent with the simulation results in Fig. 2f. A combination of the scaling $V \sim (F_L+F_{DX})/[R(\mu_o+\mu_l)]$ and Equation (3) from the main text and Equation (S10) leads to

$$V \sim \frac{F_{DX}+F_L}{R(\mu_o+\mu_l)} = \frac{\Delta T^2}{\mu_o+\mu_l}\left[D\left(\frac{3\pi^2}{48Ra^2} - \frac{4\pi^4+\pi^2}{192R^3}\right) + L\right] \tag{S11}$$

where $L = dP_c^2/(\varepsilon_0\varepsilon_d)$ is a constant, d is the thickness of the dielectric layer, μ_o and μ_l are the viscosity of oil and liquid, respectively.

To further obtain the temperature rise ΔT at the light, we first consider the heat transfer in the photothermal layer, which can be simplified to be heat conduction in a semi-infinite cylinder, and described as[3]

$$\frac{1}{b}\frac{\partial T}{\partial t} = \frac{1}{r}\frac{\partial(r\frac{\partial T}{\partial r})}{\partial x},\ 0 < r < \infty \tag{S12}$$

$$t = 0,\ T(r, t) = T_0 \tag{S13}$$

$$r = R_0,\ -\lambda\frac{\partial T}{\partial r} = q_0 \tag{S14}$$

where b, t, r, T_0, λ and q_0 denotes thermal diffusivity of photothermal film, irradiation time, the distance from any point on the surface to the laser spot center, ambient temperature, thermal conductivity of the photothermal layer and heat flux, respectively. The heat flux can be expressed as $q_0=P/(2\pi R_0 d_0)$, where d_0 is photothermal layer thickness. The analytical solution of the problem can be readily obtained as

$$T(r,t) = T_0 + \frac{P}{\lambda\pi^2 d_0}I \tag{S15}$$

$$I = \int_0^\infty \frac{1-e^{-\frac{btu}{R_0}}}{u^2} \times \frac{Y_1(u)J_0(\frac{ru}{R_0})-J_1(u)Y_0(\frac{ru}{R_0})}{J_1^2(u)+Y_1^2(u)}du \tag{S16}$$

Assuming the same temperature for the photothermal layer and the lithium niobate due to their intimate contact, the temperature rise of lithium niobate crystal at the irradiation point can be described as

$$\Delta T(r,t) = T(r,t) - T_0 = \frac{P}{\lambda\pi^2 d_0}I \tag{S17}$$

Therefore, the temperature rise at the edge of the bubble under NIR irradiation is

$$\Delta T(R_0,t) = T(R_0,t) - T_0 = \frac{P}{\lambda\pi^2 d_0}I(R_0,t) \tag{S18}$$

$$I(R_0,t) = \int_0^\infty \frac{1-e^{-\frac{btu}{R_0}}}{u^2} \times \frac{Y_1(u)J_0(u)-J_1(u)Y_0(u)}{J_1^2(u)+Y_1^2(u)}du \tag{S19}$$

Furthermore, a combination of Equation (S11) and Equation (S18), leads to

$$V \sim \frac{P^2 I^2(R_0,t)}{\lambda^2\pi^4 d_0^2(\mu_o+\mu_l)}\left(D\left(\frac{3\pi^2}{48Ra^2} - \frac{4\pi^4+\pi^2}{192R^3}\right) + L\right) \tag{S20}$$

It is clear that steady bubble moving velocity increases with the laser power but decreases with the bubble volume, which is consistent with the experimental results as shown in Fig. 1e.

Supplementary Discussion 4. Mechanism of bubble splitting

Bubble splitting between two parallel plates. As shown in Supplementary Fig. 19a, when compressed by a parallel plate, a bubble on the PESS forms a concave surface, which is subjected to a uniform outward Laplace force in the radial direction. Note that, the water contact angles at the upper (θ_{u0}) and lower (θ_{l0}) surface are equal to each other, i.e., $\theta_{u0} = \theta_{l0}$. When the NIR light irradiating left side of the bubble, as shown in Supplementary Fig. 19b, the water contact angle θ_l on the bottom surface decreases due to the generated dielectric wetting[6], which could be expressed as

$$\cos\theta_l = \cos\theta_{l0} + \frac{\varepsilon_0\varepsilon_d}{2\gamma_{LG}d}\Delta U^2 = \cos\theta_{l0} + \frac{P_c^2\Delta T^2 d}{2\gamma_{LG}\varepsilon_0\varepsilon_d} \tag{S21}$$

where θ_l and θ_{l0} are the contact angles of water and the lower surface with and without NIR irradiation, respectively, and $\Delta U = \frac{P_c\Delta T d}{\varepsilon_0\varepsilon_d}$ is the voltage drop across the dielectric layer in the vertical direction at the three-phase contact line.

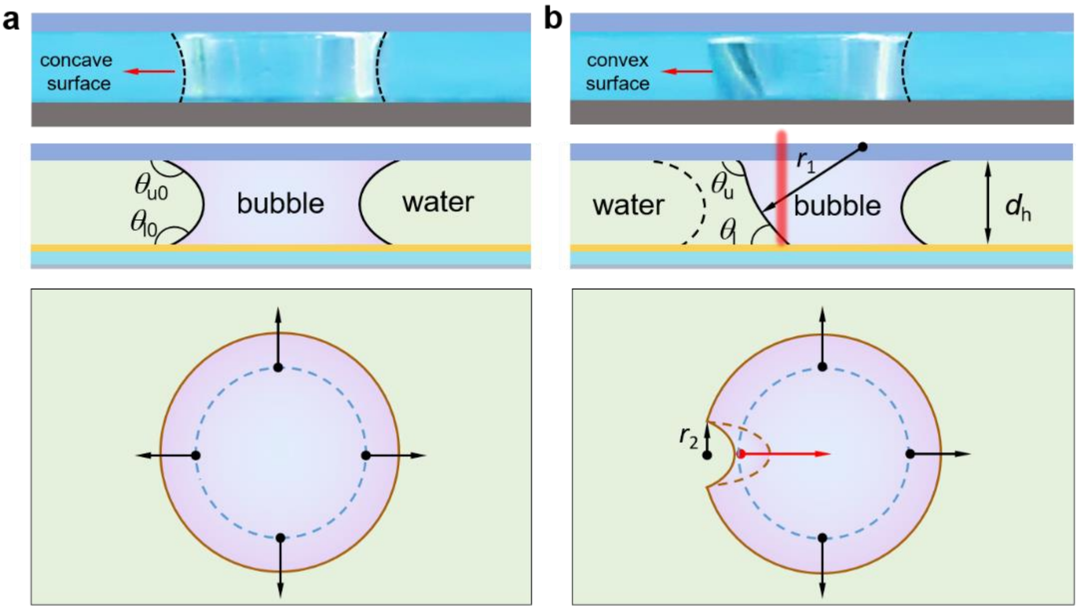

Supplementary Figure 19 | Experimental image and schematics showing the mechanism of bubble splitting between two parallel plates without (**a**) and with (**b**) NIR irradiation.

Obviously, after switching on the NIR light, a Laplace pressure towards the inside of the bubble generates at the left side due to the transformation of the concave meniscus (Supplementary Fig. 19a) to a convex one (Supplementary Fig. 19b) to split the bubble, which can be expressed as

$$P_0 = \gamma_{LG}\left(\frac{1}{r_1} + \frac{1}{r_2}\right) \tag{S22}$$

where P_0 is the pressure of the bubble, r_1 and r_2 are the principal radii of curvature, and r_2 is equal to the width of NIR spot $R_0 = 0.5$ mm. The radius of the meniscus of the bubble r_1 is geometrically related to the

local contact angles and the distance between the plates d_h, which could be expressed as

$$r_1 = \frac{d_h}{\cos\theta_u + \cos\theta_l} \tag{S23}$$

where θ_u is the contact angle of the water and the upper surface after NIR irradiation. A combination of Equations (S22) and (S23) leads to the Laplace pressure to split the bubble as

$$P_0 = \gamma_{LG}\left(\frac{\cos\theta_u + \cos\theta_l}{d_h} + \frac{1}{R_0}\right) \tag{S24}$$

It is clear from Equation (S24) that the Laplace pressure for bubble splitting increased after reducing the distance between two plates d_h, which is in line with the experimental results. Notably, the dielectrophoretic force shows little effect on the bubble splitting because it acts on the whole bubble[7,8].

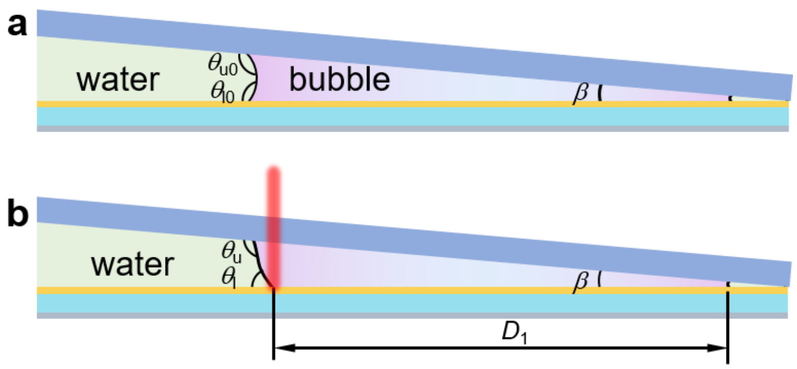

Supplementary Figure 20 | Schematics showing bubble splitting process on the PESS without (**a**) and with (**b**) NIR irradiation.

Bubble splitting between two inclined plates. When a bubble is compressed by an inclined plate on the PESS, a Laplace force is generated to drive the bubble to the tip[9] (Supplementary Fig. 20a). Under NIR irradiation, the bubble deforms to generate a Laplace pressure towards the inside of the bubble to splitting the bubble (Supplementary Fig. 20b), which can be calculated by

$$P_0 = \gamma_{LG}\left(\frac{\cos\theta_u + \cos\theta_l}{D_1 \tan\beta} + \frac{1}{R_0}\right) \tag{S25}$$

where D_1 is the bubble length in the horizontal direction, β is the angle between these two plates. It is clear that the Laplace pressure for bubble splitting increases at a reduced β, which is in line with the experimental result.

By contrast, the contact angle of a bubble compressed by a horizontal or an inclined plate on the PSS under NIR irradiation becomes smaller[1] (Supplementary Fig. 21), corresponding to a larger Laplace pressure $P_0 = \gamma_{LG}\left(\frac{\cos\theta_u + \cos\theta_l}{-d_h} + \frac{1}{R_0}\right)$ or $P_0 = \gamma_{LG}\left(\frac{\cos\theta_u + \cos\theta_l}{-D_1 \tan\beta} + \frac{1}{R_0}\right)$, respectively, towards the outside of the bubble to further extend the bubble, during which the bubble splitting is impossible.

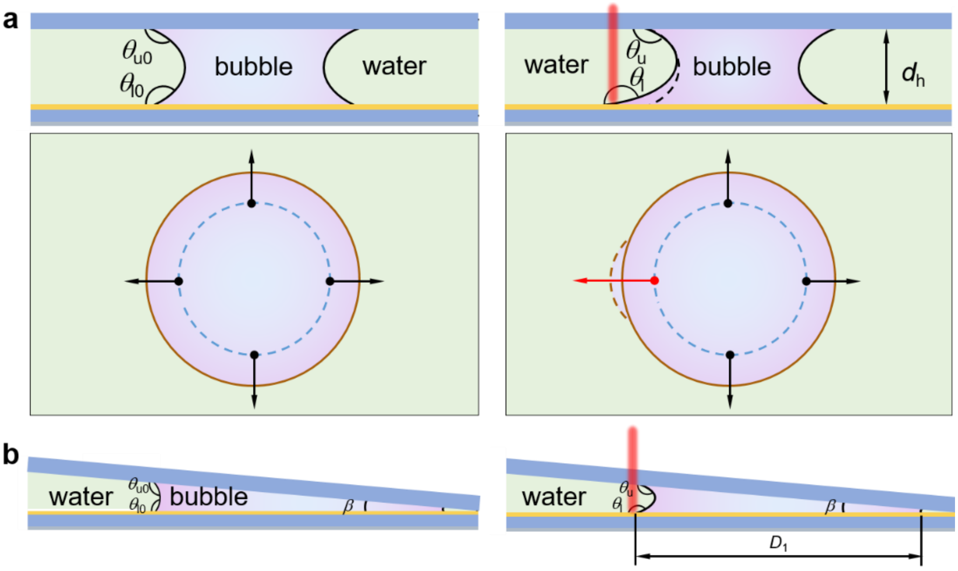

Supplementary Figure 21 | Schematics showing the force of a compressed bubble on the PSS under NIR irradiation. a Bubble compressed by a horizontal plate on the PSS. **b** Bubble compressed by an inclined plate on the PSS.

Supplementary Discussion 5. Mechanism of bubble detachment

To understand the mechanism of the bubble detachment from the PESS, we conducted the force analysis of the bubble under a static state in the vertical direction. As shown in Supplementary Fig. 22a, the forces on a bubble after irradiation are mainly composed of buoyancy force (F_B), the dielectrophoretic force along the z direction (F_{DZ}) and the capillary force (F_C)[10,11]. Among them, the direction of F_B and F_{DZ} are upward, and the direction of the vertical component of capillary force, F_{CV}, is downward. Therefore, the resultant force F_R in the vertical direction can be expressed by

$$F_R = F_B + F_{DZ} - F_{CV} = (\rho_w - \rho_g)\Omega g + F_{DZ} - 2\pi\gamma_{LG} R \sin\theta \quad \text{(S26)}$$

where ρ_g and ρ_w are the densities of air and water, respectively, and Ω is the volume of the bubble.

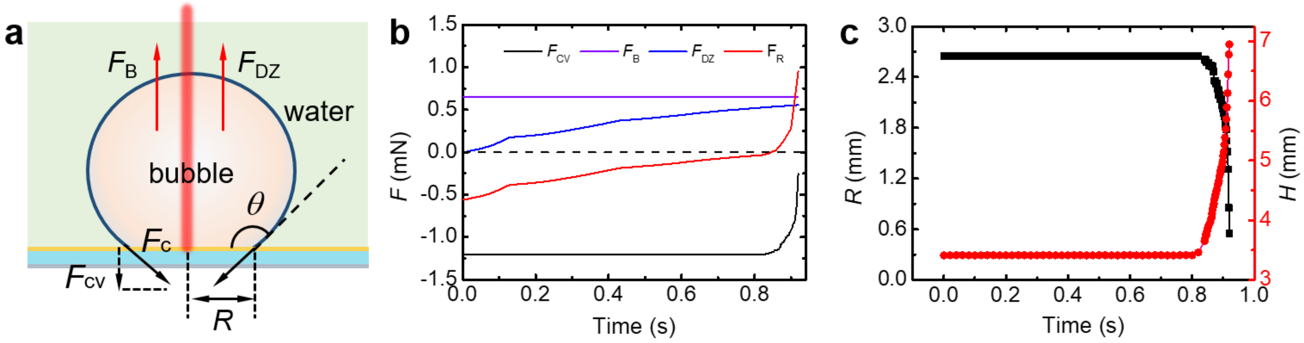

Supplementary Figure 22 | The force analysis of bubble detachment from the PESS. a Schematics showing the force analysis of bubble detaching from the PESS. **b** The variation of the forces acting on a

65-μL bubble during detachment process with time. **c** Variation of base radius *R* and bubble height *H* of a 65-μL bubble with time under the irradiation of 500-mW NIR light. Source data are provided as a Source Data file.

Supplementary Fig. 22b shows the variation of the forces acting on a 65-μL bubble during detachment process with time. When the NIR light initially irradiates the PESS, F_C is greater than the upward force on the bubble, and the bubble remains essentially unchanged. As the light exposure time increases, F_{DZ} gradually increases. At around 0.83 s, F_R on the bubble becomes greater than 0, which causes *R* to decrease rapidly while *H* increases rapidly (Supplementary Fig. 22c), as well as a rapid decrease in the adhesion force on the bubble. Ultimately, the bubble detaches from the surface at ~ 0.92 s. It is clear that the increase of dielectrophoretic force with time is the main cause of bubble detachment. Therefore, increasing the power of NIR laser to increase the dielectrophoretic force can make it easier for the bubbles to escape from the PESS. Furthermore, increasing the bubble volume to increase the upward buoyancy of the bubble also contributes to the rapid bubble escape.

Supplementary References

1. Dai, L., Ge, Z., Jiao, N. & Liu, L. 2D to 3D manipulation and assembly of microstructures using optothermally generated surface bubble microrobots. *Small* **15**, 201902815 (2019).
2. Chen, C. et al. Remote photothermal actuation of underwater bubble toward arbitrary direction on planar slippery Fe_3O_4-doped surfaces. *Adv. Funct. Mater.* **29**, 19047 (2019).
3. Li, W., Tang, X. & Wang, L. Photopyroelectric microfluidics. *Sci. Adv.* **6**, eabc1693 (2020).
4. Wang, F. et al. Light-induced charged slippery surfaces. *Sci. Adv.* **8**, eabp9369 (2022).
5. Wang, F. et al. Light control of droplets on photo-induced charged surfaces. *Natl. Sci. Rev.* **10**, nwac164 (2023).
6. Peykov, V., Quinn, A. & Ralston, J. Electrowetting: a model for contact-angle saturation. *Colloid Polym. Sci.* **278**, 789-793 (2000).
7. Jones, T. B. Dielectrophoretic force calculation. *J. Electrost.* **6**, 69-82 (1979).
8. Zhao, Y. & Cho, S. K. Micro air bubble manipulation by electrowetting on dielectric (EWOD): transporting, splitting, merging and eliminating of bubbles. *Lab on a Chip* **7**, 273-280 (2007).
9. Prakash, M., Quere, D. & Bush, J. W. Surface tension transport of prey by feeding shorebirds: the capillary ratchet. *Science* **320**, 931-934 (2008).

10. Yan, R., Pham, R. & Chen, C. L. Activating bubble's escape, coalescence, and departure under an electric field effect. *Langmuir* **36**, 15558-15571 (2020).

11. Chen, F., Peng, Y., Song, Y. Z. & Chen, M. EHD behavior of nitrogen bubbles in DC electric fields. *Exp. Therm. Fluid Sci*. **32**, 174-181 (2007).
