# Supplementary material for: Versatile bubble maneuvering on photopyroelectric slippery surfaces
Source: Nat Commun. 2023 Oct 3;14:6158. doi: 10.1038/s41467-023-41918-y (PMC10547833; doi:10.1038/s41467-023-41918-y)
Supplement: Supplementary file 3 — Description of Additional Supplementary Files [file 41467_2023_41918_MOESM3_ESM.docx]

**Description of Additional Supplementary Files**

**Supplementary Movies 1**

The NIR-induced transport of a 35-L bubble on the PESS

**Supplementary Movies 2**

The reverse buoyancy movement of bubbles on the PESS

**Supplementary Movies 3**

The asymmetric bubble deformation on the PESS under the NIR irradiation

**Supplementary Movies 4**

A 35-L bubble was driven when the NIR light is irradiated at 3 mm from the left end of the bubble

**Supplementary Movies 5**

Arbitrary trajectory transport of bubbles on the PESS

**Supplementary Movies 6**

Bubble splitting on the PESS

**Supplementary Movies 7**

The detachment of a 65-L bubble on the PESS under the NIR irradiation

**Supplementary Movies 8**

Multi-functional integration of bubble manipulation on the PESS

**Supplementary Movies 9**

Bubble microrobots
